# A Case of Ganser Syndrome Presenting as a Stroke Alert: Fact or Factitious?

**DOI:** 10.7759/cureus.71736

**Published:** 2024-10-17

**Authors:** Yulong H Stokes, Mohammad Jahangiri, Juan G Ochoa

**Affiliations:** 1 Department of Neurology, University of South Alabama College of Medicine, Mobile, USA; 2 Department of Neurology, Univeristy of South Alabama College of Medicine, Mobile, USA

**Keywords:** conversion disorder, dissociative disorder, functional neurological disorder, ganser syndrome, partial epilepsy

## Abstract

Ganser syndrome is a rare and historically controversial disorder characterized by approximately correct (but not quite correct) answers to simple questions (called vorbeigehen or vorbeireiden), visual and auditory hallucinations, and “clouded consciousness” or encephalopathy. These symptoms usually co-occur with various functional neurological symptoms.

We present the case of a 53-year-old man who arrived as a stroke alert with sudden-onset left-sided weakness and numbness, one day after being reported to police by a neighbor for indecent exposure. During his emergency department evaluation, the examiner noticed that he exhibited an inconsistent bedside mental status examination, responding with approximate but not quite correct answers that indicated awareness of his own thought content, in addition to reporting nondescript hallucinations. He had one previous hospitalization in our records with a similar presentation but without these characteristic mental status changes. He serves as his own control, demonstrating that fear of punishment or legal action was necessary to elicit a presentation of Ganser syndrome in his case.

## Introduction

In 1897, Saxon psychiatrist Sigbert Josef Maria Ganser studied a fascinating phenomenon in three prisoners whose symptoms could be encompassed by the presence of four primary characteristics: approximation of answers, visual and auditory hallucinations, clouding of consciousness, and conversion symptoms. Of these four characteristics, the most striking is the approximation of answers when asked simple questions, defined in German as vorbeigehen ("to pass by the point") or vorbeireiden ("to talk beside the point") [1-4]. For instance, when asked how many legs a cow has, the answer may be “three” or “six.” When pointing to a chair and asking the patient to name the object, the answer may be “couch” [1,2]. Hence, the eponym of Ganser syndrome was born. Notably, despite these characteristics being considered the hallmarks of Ganser syndrome, patients frequently do not present with all four symptoms. In the three cases studied by Ganser himself, all experienced rapid self-resolution as well as amnesia of the events following the resolution of their symptoms [1]. Ganser syndrome is often associated with stressful events such as incarceration [1-3]. In fact, it was initially thought to be a “prison psychosis” [1]. Nevertheless, Ganser syndrome remains an extremely rare diagnosis, with only 117 documented cases in the literature from 1897 to 2022, 10 of which were described in children and adolescents. Based on this limited information, Ganser syndrome seems to affect primarily male minorities in their early thirties who have either been incarcerated or undergone another source of significant stress [2].

The etiology, pathophysiology, diagnosis, and treatment of Ganser syndrome are poorly understood, as the criteria for establishing the diagnosis are vague [1,2]. It shares similarities with dissociative disorders, conversion disorder, psychosis, malingering, and factitious disorder [2]. Treatment options are largely supportive, as Ganser syndrome frequently self-resolves without intervention; however, pharmacotherapy such as antipsychotics or antidepressants has also been trialed with mixed results [1-3, 5-8]. Patients typically present with waxing and waning levels of consciousness and may at times appear to be malingering, thus potentially confounding the true diagnosis [1]. The syndrome does not seem to correlate with any brain imaging findings, and there is no standardized evaluation due to its low prevalence [1,2,4,6,9].

## Case presentation

We present the case of a 53-year-old male who presented to the emergency department with complaints of sudden-onset left hemibody arm and leg weakness sparing the face, without neck pain, radicular pain, or urinary incontinence. At baseline, he reported weakness of his left leg described as a foot drop; during his epilepsy monitoring unit (EMU) admission, his baseline was documented as four out of five for hip flexion, four out of five for knee flexion and extension, one out of five for dorsiflexion, and five out of five for plantarflexion (Medical Research Council Scale). Prior to arrival, the patient collapsed and hit his head on the floor at home, followed by shivering for five minutes, as witnessed by his wife. While initially described as minimally interactive, the patient was able to respond to his wife to some degree during the episode, stating, “I feel cold.” He expressed subjective confusion and was transferred to the hospital as a stroke alert. Notably, the day prior to admission, the patient was reported to and questioned by police after exposing himself to his female neighbor. After being evaluated by neurology in the emergency department, he expressed suicidal ideation to his wife, which was eventually reported to emergency department staff.

Past medical and surgical history

The patient’s medical history was significant for left subependymoma status post-surgical resection via transcallosal-ventricle approach two years ago, cardiogenic syncope with bradycardia, psychogenic nonepileptic seizures, and left temporal lobe epilepsy. He reported no family history of seizures. He took lamotrigine 200 mg nightly for seizures. He had several relevant hospitalizations, upon which we elaborated below.

Several months prior to admission, the patient was admitted to the epilepsy monitoring unit (EMU) for a history of seizure-like events. During his stay, the physician observed that the semiology of the nonepileptic episodes involved brief, 15 to 20 seconds of staring episodes, sometimes accompanied by left hand posturing as if stroking the chin, followed by a brief period of subjective confusion and decreased interactivity. One instance of a questionable left temporal seizure pattern was captured on electroencephalogram (EEG), which correlated with the same semiology of chin stroking with the left hand. Unfortunately, a record of the EEG could not be obtained for publication in this report due to technical problems with the storage server.

Three years ago, the patient presented to the hospital after a motor vehicle accident with very similar complaints of dense left hemibody numbness and weakness one year prior to his subependymoma resection. Per our chart review, it was documented and reported that the accident was minor, with minimal injury to the patient, wife, and son. The patient did not appear to lose consciousness but reported hitting his head. Per emergency medical services (EMS), the patient had a Glasgow Coma Scale (GCS) of 15 and was interactive prior to arrival, initially able to give a good history. However, fifteen minutes later, during EMS transport to the hospital, he reportedly appeared to be “dazed” and poorly interactive prior to developing his left-sided weakness and numbness. He was evaluated by the trauma team without any major injuries or fractures. A stroke alert was called, and neurology evaluated the patient. The non-contrast head computed tomography (CT) was normal, and the CT angiogram of the head and neck showed no large vessel occlusion or significant stenosis. The magnetic resonance imaging (MRI) of the brain was unremarkable except for a suspected subependymoma approximately two centimeters in diameter noted in the left lateral ventricle, and the MRI of the cervical spine was similarly unrevealing. Neurology and neurosurgery provided the patient with a final diagnosis of spinal cord injury without radiographic abnormality (SCIWORA) prior to discharge. The patient's mass was resected approximately one year later due to growth, and pathology revealed a WHO Grade I subependymoma.

Examination

During the mental status examination, the patient was sufficiently alert and aware enough to recount most of the presenting history (as corroborated by his wife) and provide an up-to-date medication list complete with dosages. When asked pointed questions meant to evaluate cortical function, the patient provided approximate answers. For instance, when evaluating his orientation to location, the patient responded that he was at the dentist’s office. Immediately suspicious, the examiner asked the patient why he thought he was at the dentist’s office. The patient briskly responded by pointing out the presence of the mounted operating room lights in the trauma bay. He incorrectly identified the present city as “Paducah, Kentucky.” He reported the month and year as November 2000 (the patient presented in October 2023). When asked how many legs a horse has, he responded, “An octopus has eight legs.” He was able to perform simple math, such as "5 + 4 = 9." He could repeat phrases appropriately and fluently, follow three-step commands with laterality, and name two out of three objects correctly on examination. When asked if he was experiencing auditory or visual hallucinations, he simply responded “yes” to both but remained silent on follow-up questioning regarding the content of his hallucinations.

Cranial nerve examination was notable for completely absent right hemifacial sensation. The motor examination was significant for giveaway weakness, but the patient was able to be coached to 4 out of 5 strength (Medical Research Council Scale) compared to the contralateral side. No meningismus was noted. However, the left leg was only able to be coached up to 3 out of 5 strength at the hip and knee, with 0 out of 5 strength on dorsiflexion and plantar flexion. The patient also reported completely absent sensation in the left arm and left leg to light touch and light pinprick but frankly withdrew to surreptitious nail bed pressure at the left hand. Deep tendon reflexes were normal except for 3+ reflexes at the bilateral patellae. Hoffman sign was negative, and plantar responses were down-going bilaterally.

Labs and imaging

**Figure 1:**
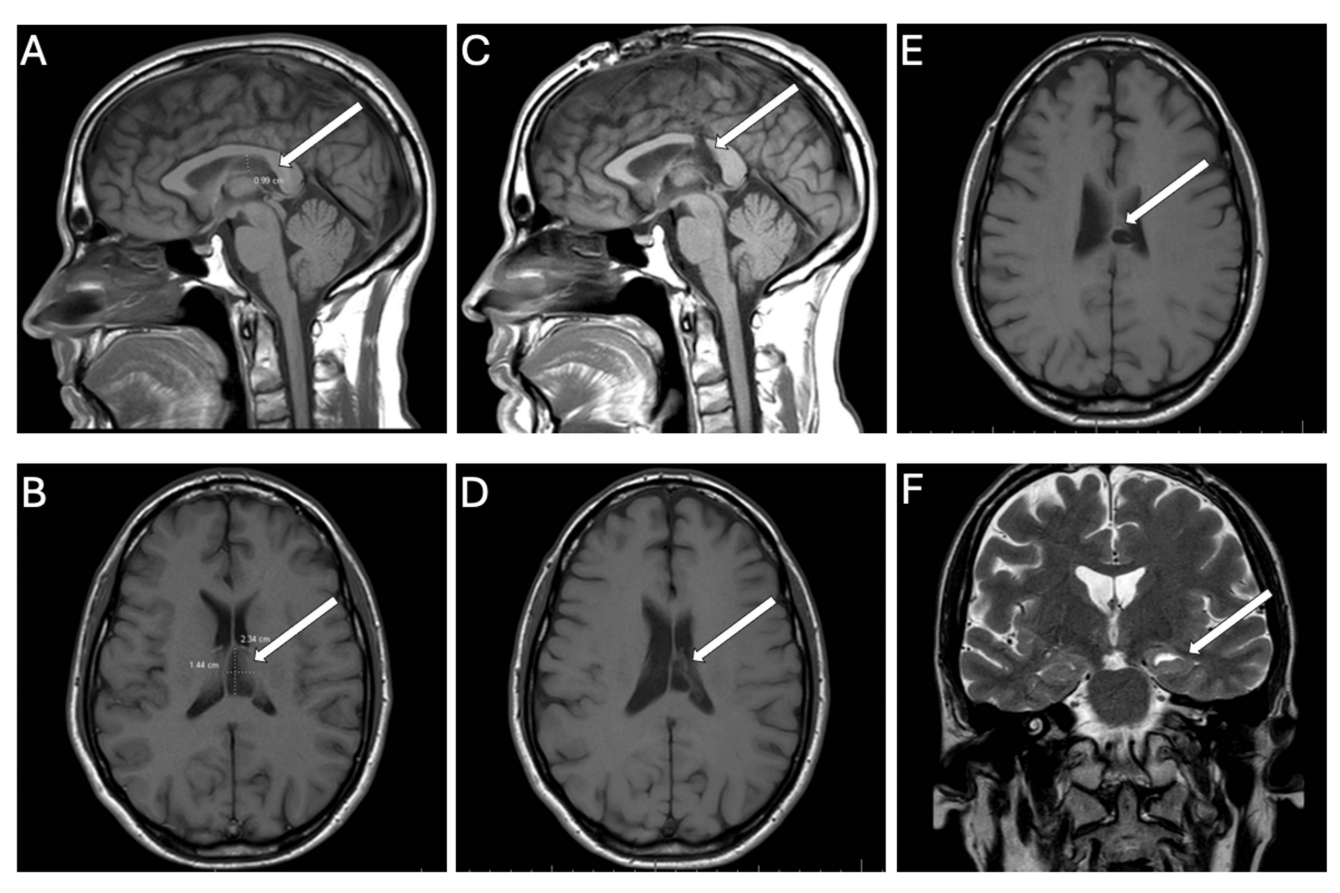
Magnetic resonance imaging (MRI) of the patient’s brain before and after subependymoma resection three years prior to presentation. Panels A and B are T1 sagittal and axial sections demonstrating the location of the initial Grade I subependymoma in the left third ventricle (marked by white arrows). Panels C, D, and E are T1 sagittal and axial sections at the time of hospital presentation demonstrating encephalomalacia from postsurgical changes and external ventricular drain tract years after resection (marked by white arrows). Panel F is a T2 sequence showing volume loss in the left hippocampus consistent with mild left-sided mesial temporal sclerosis (marked by a white arrow).

On arrival, the patient underwent a stroke evaluation, including a non-contrasted CT head and a CT angiogram of the head and neck, which were both normal. Overnight, the patient underwent an MRI of the brain and an MRI of the cervical spine without contrast, both of which were notable for no acute findings (including no DWI changes) compared to prior imaging. The MRI of the brain showed no overt signs of central nervous system inflammation, white matter changes, or acute stroke, with stable postsurgical changes from subependymoma resection, as well as mild frontal encephalomalacia from an external ventricular drain (EVD) tract. Of note, the patient had a known finding of medial temporal sclerosis on the left side (see Figure 1).

The patient was evaluated for potential etiologies of acute or subacute encephalopathy with behavioral changes. The patient was noted to be afebrile with stable vital signs throughout his hospitalization. Cell counts and chemistries were normal. Labs were only significant for benzodiazepines on the urine drug screen (from intravenous lorazepam administered by emergency medical services (EMS) prior to arrival). Cerebrospinal fluid was mostly unremarkable, with a slightly elevated protein level of 57 mg/dL. A broad-spectrum panel for paraneoplastic and autoimmune etiologies of encephalopathy was sent out and returned negative (see Table 1). Unfortunately, the broad-spectrum infectious polymerase chain reaction (PCR) panel, including the herpes simplex virus, was not sent at this time due to low suspicion for active infection. Blood and urine cultures were negative for bacterial growth.

**Table 1:** Cerebrospinal Fluid (CSF) Testing All pertinent testing was performed on the CSF. The second column lists the normal range for the studies, while the third column presents the patient results for the studies. Abnormal values are bolded. The autoimmune/paraneoplastic encephalopathy panel includes antibody testing for N-methyl-D-aspartate (NMDA) receptor, alpha-amino-3-hydroxy-5-methyl-4-isoxazole propionic acid (AMPA) receptor, amphiphysin, antiglial nuclear antibody-1 (AGNA-1), antineuronal nuclear antibody types 1, 2, and 3 (ANNA-1, ANNA-2, ANNA-3), contactin-associated protein-like 2 (CASPR2), collapsin response-mediator protein-5 (CRMP5), dipeptidyl-peptidase-like protein-6 (DPPX), gamma-aminobutyric acid B (GABA-B), glutamic acid decarboxylase 65-kilodalton isoform (GAD65), glial fibrillary acidic protein (GFAP), immunoglobulin LSAMP/OBCAM/Neurotrimin family member 5 (IgLON5), leucine-rich glioma inactivated 1 (LGI1), metabotropic glutamate receptor 1 (mGluR1), neurochondrin, neuronal intermediate filament (NIF), purkinje cytoplasmic antibody types 1, 2, and Tr (PCA-1, PCA-2, PCA-Tr), and septin-7. WBC: white blood cell, RBC: red blood cell.

Investigation	Patient Value	Normal Value
WBC	2 (1 mononuclear, 1 polymorphonuclear)	0-5 cells/mcL
RBC	0	0-2 cells/mcL
Glucose	62	40-70 mg/dL
Protein	57	15-45 mg/dL
CSF autoimmune/paraneoplastic encephalopathy panel	Undetectable for antibodies against NMDA receptor, AMPA receptor, amphiphysin, AGNA-1, ANNA-1, ANNA-2, ANNA-3, CASPR2, CRMP-5, DPPX, GABA-B receptor, GAD65, GFAP, IgLON5, LGI1, mGluR1, neurochondrin, NIF, PCA-Tr, PCA-1, PCA-2, septin-7.	

Subsequent hospital course

The patient improved over the course of the next few days, stating that he had more or less no confusion the day after admission. Given that he had expressed suicidal ideation, he was placed on one-to-one observation, and psychiatry was consulted. Over the next two days of hospitalization, he denied any further suicidal or homicidal ideation. Psychiatry diagnosed him with an unspecified depressive disorder and recommended starting mirtazapine 15 mg nightly with outpatient follow-up. Psychiatry deemed him safe for discharge at that point.

However, due to continued left hemibody weakness as well as delays in obtaining MRI scans, he was held in the hospital for two more days. No further events or changes in examination occurred during that time. He was eventually discharged home with instructions to follow up in the epilepsy clinic. His lamotrigine was increased from 200 to 250 mg daily. He was set up with home health physical therapy.

## Discussion

Historically, the classification of Ganser syndrome has been controversial. Although it was most recently categorized in the Diagnostic and Statistical Manual of Mental Disorders, Fourth Edition (DSM-IV) as a dissociative disorder, it has since been removed from the Fifth Edition. The International Classification of Diseases, Eleventh Revision (ICD-11), classifies Ganser syndrome as a dissociative disorder and a form of conversion disorder [2]. Despite its inclusion in these texts, the criteria for establishing the diagnosis of Ganser syndrome are vague [1,2]. Several reported cases of Ganser syndrome are associated with focal neurologic deficits, including hemiparesis, numbness, difficulty walking, and inability to see [2,5,8]. Executive dysfunction to some degree, including hypomimia, acalculia, anosognosia, and disorientation, is present in most cases in contemporary literature [1-10]. Although typically self-resolving within a few weeks, one case of Ganser syndrome reported by Wincewicz et al. was found to be chronic and recurrent, occurring twice in a prisoner’s lifetime and lasting greater than two years each instance [2]. Given the rarity of the diagnosis, other disease processes must be considered when evaluating a patient with suspected Ganser syndrome. Among the differential diagnoses for Ganser syndrome are malingering, factitious disorder, psychosis, dementia, pseudodementia, or a combination of the above. The etiology of Ganser syndrome is also unclear due to very few cases and controlled studies, as it seems to occur with other pathologies, including traumatic brain injury, depression, anxiety, posttraumatic stress disorder (PTSD), and stroke. Whether these are risk factors for Ganser syndrome or simply coexist with it is yet to be elucidated [1-5]. Furthermore, Ganser syndrome is a diagnosis of exclusion [7].

In one study described by Dalfen et al., the authors performed a retrospective chart review of 513 patients seen over one year in an outpatient traumatic brain injury clinic. Of these patients, four were diagnosed with Ganser syndrome, all of whom experienced post-concussion disorder and mild posttraumatic amnesia without retrograde amnesia. Three of these four patients were found to have acute stress disorder (ASD), with one case progressing to PTSD. Both ASD and PTSD have been linked to dissociative symptoms, of which Ganser syndrome is a subtype. Only one patient pursued litigation, arguing against the notion that Ganser syndrome is a form of malingering [4]. In an epidemiological study conducted by Tsoi at Woodbridge Hospital in Singapore, he identified 21 suspected cases of Ganser syndrome, of which 10 were sufficient for diagnosis of Ganser syndrome [3,10]. Interestingly, answer approximation was similarly preserved among Chinese, Indian, and Malay patients when compared with English Europeans, highlighting that this phenomenon persists despite cultural differences [10]. Tsoi believed the patients to be on the continuum of hysteria and malingering rather than psychosis, although the consensus from more modern studies supports Ganser syndrome as a dissociative and/or conversion disorder, consistent with its DSM-IV and ICD-11 classification [1,2,5,10]. Ganser syndrome could, on the other hand, be a mix of a dissociative and factitious disorder, as the two are not mutually exclusive [5].

The patient presented with several typical features and risk factors for Ganser syndrome, including a recent history of involvement with law enforcement, fear of legal action, approximation of answers, vague hallucinations, questionable head trauma history, prior brain tumor status post-resection, and major depressive disorder. A few days prior to hospitalization, the patient had been reported to the police for indecent exposure to a neighbor, after which the neighbor physically assaulted the patient. The patient had to present to the police station to give a statement. He denied headache, neck pain, neck stiffness, fevers, and chills at the time but did endorse anxiety. The proximity of his law enforcement encounter to his symptom onset implies that this major stressor could have predisposed our patient to a psychogenic episode leading to Ganser syndrome, which is consistent with the earliest cases of Ganser syndrome described in prisoners [1,2]. Furthermore, he had a similar presentation with left hemibody weakness and numbness several years prior without a Ganser-like presentation. On the other hand, the trauma sustained from his fall or previous head trauma could also predispose him to Ganser syndrome [2-4]. Whether his medical and psychiatric history predisposed him to Ganser syndrome or merely co-occurred with it is not fully understood. During his hospitalization, organic causes of his behavioral abnormalities, unilateral weakness, and numbness-such as encephalopathy, stroke, and seizure-were ruled out, suggesting a psychogenic etiology of his left-sided weakness and approximation of answers with otherwise preserved memory. Todd’s paresis was unlikely, given that possible structural etiologies of epileptic seizures were in the left hemisphere. At subsequent clinic visits, the patient’s condition seemed to spontaneously resolve from his hospitalization, except for left foot drop. No nerve conduction studies or electromyograms have been performed yet to date.

There are no universal diagnostic criteria for Ganser syndrome, and the terminology previously used by Dr. Ganser would now be considered outdated and vague [1,2,10]. The distinction between answers that are nonsense, approximately correct, and answers that capture the essence of a question can be subjective [2]. However, the authors believe that the presence of a dissociative disorder is a necessary factor for the presentation of Ganser syndrome. In this case, the patient has a long-standing history of functional neurological disorder and psychogenic nonepileptic seizures. It appears from this patient’s case that a key factor predisposing someone to a Ganser syndrome presentation may in fact be fear of punitive action by a third party, including fear of legal action or punishment.

## Conclusions

We believe that this case of Ganser syndrome falls within the spectrum of dissociative disorders, with additional diagnoses of psychogenic non-epileptic seizures and functional neurological disorder. We cannot fully exclude the possibility of a factitious disorder, whether superimposed or as a standalone diagnosis.

The case is somewhat confounded by his history of questionable head trauma, grade I subependymoma in the left lateral ventricle status post-resection, and left temporal seizures; however, these may be considered controlled to some degree, given that he had a very similar hospitalization several years prior to his brain tumor resection, without fear of legal problems to motivate the patient to malinger. Thus, fear of impending incarceration, legal action, or punishment imposed by a third party seemed to be necessary to draw out a presentation consistent with Ganser syndrome in the patient’s case.
